# Increasing Representativeness in the *All of Us* Cohort Using Inverse Probability Weighting

**DOI:** 10.1101/2024.10.02.24314774

**Published:** 2024-10-02

**Authors:** Manoj S. Kambara, Shivam Sharma, John L. Spouge, I. King Jordan, Leonardo Mariño-Ramírez

**Affiliations:** 1 National Institute on Minority Health and Health Disparities, National Institutes of Health, Bethesda, Maryland, USA; 2 IHRC-Georgia Tech Applied Bioinformatics Laboratory, Atlanta, Georgia, USA; 3 School of Biological Sciences, Georgia Institute of Technology, Atlanta, Georgia, USA; 4 National Library of Medicine, National Institutes of Health, 8600 Rockville Pike, Bethesda, Maryland, USA

## Abstract

Large-scale population biobanks rely on volunteer participants, which may introduce biases that compromise the external validity of epidemiological studies. We characterized the volunteer participant bias for the *All of Us* Research Program cohort and developed a set of inverse probability (IP) weights that can be used to mitigate this bias. The *All of Us* cohort is older, more female, more educated, more likely to be covered by health insurance, less White, less likely to drink or smoke, and less healthy compared to the US population. IP weights developed via comparison of a nationally representative database eliminated the observed biases for all demographic and lifestyle characteristics and reduced the observed disease prevalence differences. IP weights also impact genetic associations with type 2 diabetes across diverse ancestry cohorts. We provide our IP weights as a community resource to increase the representativeness and external validity of the *All of Us* cohort.

## Introduction

The aim of epidemiological studies is to study the risk and occurrence of disease in whole populations, and to do this, studies rely on samples that are intended to be representative of a larger target population. Representative sampling of study participants ensures that results derived from samples are externally valid, i.e. that they apply to the target population. The *All of Us* Research Program is a study that aims to build a large and diverse sample of the US population.^1,2^ The *All of Us* program collects participant data on demographics, social determinants of health, genetic factors, and health outcomes.^2–4^ The participants that make up the *All of Us* cohort are volunteers, and because of this, the cohort is not nationally representative. Due to the lack of representativeness of the *All of Us* cohort, and the potential for volunteer participation bias, results of epidemiological studies of the *All of Us* cohort may not be externally valid with respect to the US population.^5,6^

This problem has been well outlined in other large population biobank studies,^5,6^ most notably the UK Biobank (UKBB).^7^ The UKBB cohort was built similarly to the *All of Us* program, by recruiting volunteers to contribute data, and thus is not nationally representative.^8^ Schoeler et. al. addressed the issue of national representativeness within the UKBB using inverse probability (IP) weighting.^9^ Using the IP weighting method, they were able to weight a sample of the UKBB such that it better matched the UK population and demographic makeup. They further showed that the participation bias in the UKBB affected downstream genomic studies on UKBB individuals.

The extent of participation bias in the *All of Us* participant cohort, and the extent to which it reflects the US population, have not yet been systematically measured. The first aim of this study was to evaluate the representativeness and potential participation bias in the *All of Us* cohort by quantifying differences between *All of Us* participants and the US population for a variety of demographic, social, lifestyle, and health-related characteristics. The second aim of this study was to develop and apply IP weights to increase the representativeness of the *All of Us* participant cohort, thereby supporting the external validity of epidemiological studies of the cohort. The third aim of the study was to apply IP weights to genome-wide association studies (GWAS) of *All of Us* participants to evaluate how they may change genetic associations within and between ancestry groups.

## Results

### Sample Makeup

For a nationally representative sample to compare the *All of Us* cohort against, we used data from the 2017 – March 2020 National Health and Nutrition Examination Survey (NHANES) run by the Centers for Disease Control (CDC).^10^ We started with a total NHANES sample of n=15,560 participants, with data for nine demographic, social, and lifestyle characteristics common with *All of Us* participant data (Supplementary Figure 1). After exclusion of NHANES individuals with missing data among these nine variables, and restricting the age range, we ended with a final sample of n=7,430 participants. For the *All of Us* dataset, we started with an initial cohort of n=379,454 participants, with data for at least one of the nine demographic, social, and lifestyle variables (Supplementary Figure 2). After exclusion of *All of Us* participants with missing data among the nine variables, and restricting the age range, we ended with a final sample of n=312,210. Details on the study cohort construction, and the harmonization of variables between the NHANES and All of Us cohorts, can be found in Methods section and Supplementary Table 1.

### *All of Us* is different from the US population

We found that the *All of Us* participant cohort differed substantially from the US population. Compared to participants from the nationally representative NHANES cohort, *All of Us* participants differed for all nine characteristics considered here. *All of Us* participants are older, more female, more educated, more US born, less married, more likely to be covered by health insurance, smoke less frequently, and drink less alcohol than the US population. The differences between the nationally representative NHANES sample and the *All of Us* cohort can be seen in Table 1.

### Inverse probability (IP) weights for *All of Us*

**Figure 1.**
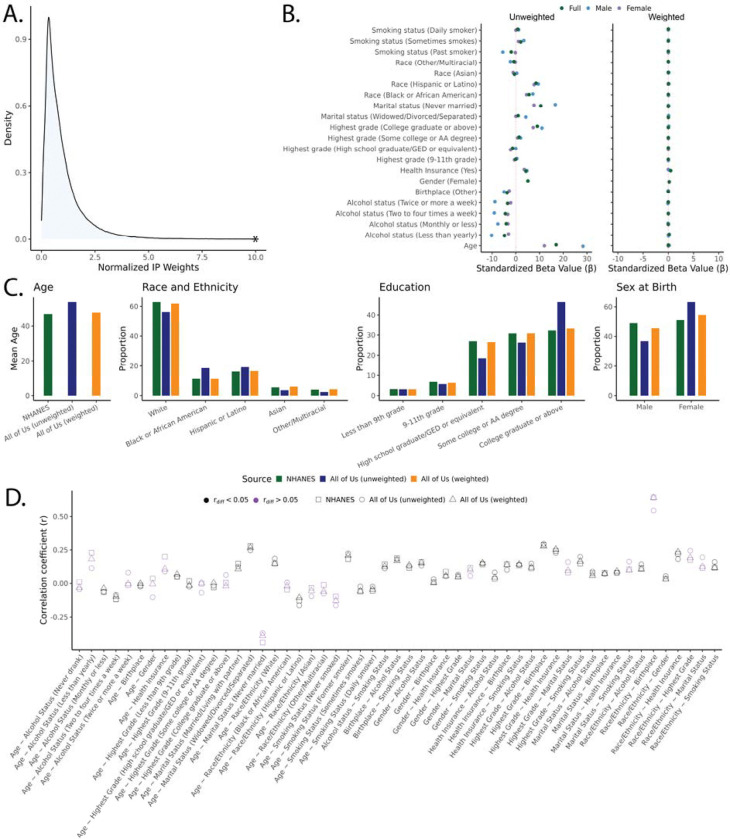
Inverse probability (IP) weights for the *All of Us* cohort. (A) Distribution of normalized IP weights developed for *All of Us* participants. (B) Beta coefficients of auxiliary variables prior to and after applying IP weights to univariate LASSO regression models. (C) Participant means and proportions for age, race and ethnicity, education, and sex for the NHANES (green), *All of Us* unweighted (blue), and *All of Us* weighted (orange) cohorts. Correlation coefficients between participant variables included in the model. (D) Correlation coefficients for all pairs of variables are shown for NHANES (squares), *All of Us* unweighted (circle), and *All of Us* weighted (triangle). Purple color indicates differences in correlation coefficients between NHANES and *All of Us* >0.05 prior to IP weighting.

To calculate IP weights for *All of Us* participants, we developed a LASSO regression model that predicts the *All of Us* participation probability based on the nine harmonized variables for the *All of Us* cohort and the NHANES cohort. The IP weights model also included all possible two-way interactions between the nine variables. Using the participation probabilities predicted from this model, we derived IP weights for *All of Us* participants, with a range of values from 0.01 to 28.74. Participants with characteristics that are overrepresented in the *All of Us* cohort compared to the US population are assigned lower IP weights, whereas participants with underrepresented characteristics are assigned higher IP weights. The process of variable selection for the LASSO regression IP weight model can be found in Supplementary Figure 3, and Figure 1A shows the distribution of the normalized IP weights derived for *All of Us* individuals. To evaluate the performance of IP weights, we first tested if the weights reduced the effect of the nine participant variables on cohort participation within a univariate regression model. Figure 1B shows that the effects of all variables became non-significant when IP weights are included in the model, showing that the weighting was effective. We found that IP weighting of the *All of Us* cohort effectively recovered US population means and proportions for all variables included in the model. Figure 1C shows the effect of IP weighting on age, race and ethnicity, gender, and education, and the effect of IP weighting on the other variables included in the IP weight model can be seen in Supplementary Table 2 and Supplementary Figure 4.

The inclusion of interaction terms in the *All of Us* participation regression model allowed us to accommodate the correlation structure in the data when estimating IP weights. This can be seen when participation bias is quantified as the difference between the pairwise correlation coefficients $\left(r_{X.Y}\right)$ for participant variables calculated using the NHANES or the *All of Us* cohorts $\left[r_{\mathrm{diff}}=NHANES\left(r_{X.Y}\right)-AllofUs\left(r_{X.Y}\right)\right]$ (Figure 1D). We found IP weighting systematically reduced the differences in correlation coefficients ($r_{\mathrm{diff}}$) between pairs of variables in NHANES versus *All of Us*.

**Figure 2.**
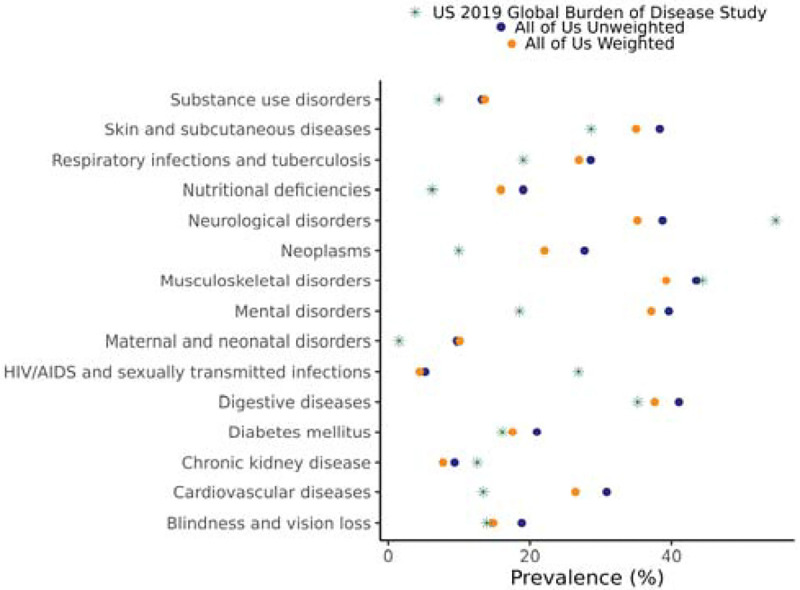
Disease prevalence for *All of Us* cohort and the US population. Disease prevalence estimates for 15 broad disease categories are shown for the US population, estimated from GBD2019 data (green stars), the unweighted *All of Us* cohort (blue circles), and the weighted *All of Us* cohort (orange circles).

Next, we found that disease prevalence estimates for the *All of Us* cohort were brought closer to US population estimates after IP weighting. We compared US population prevalence values for 15 disease categories, taken from the 2019 Global Burden of Disease Study (GBD2019)^11^, to the prevalences within the *All of Us* cohort. Figure 2 shows disease prevalence estimates from GBD2019 (considered nationally representative) compared to prevalence estimates from unweighted and weighted versions of the *All of Us* cohort. We found that the *All of Us* cohort showed a higher prevalence than the US population for 11 out 15 disease categories, and IP weighting brought these prevalence values closer to that of the US population. Nevertheless, the weighted *All of Us* cohort disease prevalence values remain higher than US population estimates for 9 of the disease categories. IP weighting brings All of Us cohort prevalence values in line with US population estimates for diabetes mellitus and blindness and vision loss. The same patterns can be seen when males and females are analyzed separately (Supplementary Figures 5 and 6).

### Type 2 diabetes genome-wide association analysis (GWAS)

We found that the diabetes mellitus prevalence estimate for the *All of Us* cohort was moved closer to the US population estimate using IP weighting and thus sought to evaluate the effect of IP weighting on genetic associations with type 2 diabetes. The unweighted prevalence of type 2 diabetes within the *All of Us* cohort is 23.2%, and the weighted prevalence is 19.7%, closer to the US population prevalence estimate of 16.1%. We also analyzed how IP weighting affected type 2 diabetes disparities between African and European ancestry groups (Supplementary Table 3). In the unweighted *All of Us* cohort, the African ancestry group of participants (30.0%) has 11.1% higher type 2 diabetes prevalence than the European ancestry group (18.9%). This disparity was reduced by nearly half (to 6.0%) in the weighted *All of Us* cohort (African=23.8% and European=17.8%).

**Figure 3.**
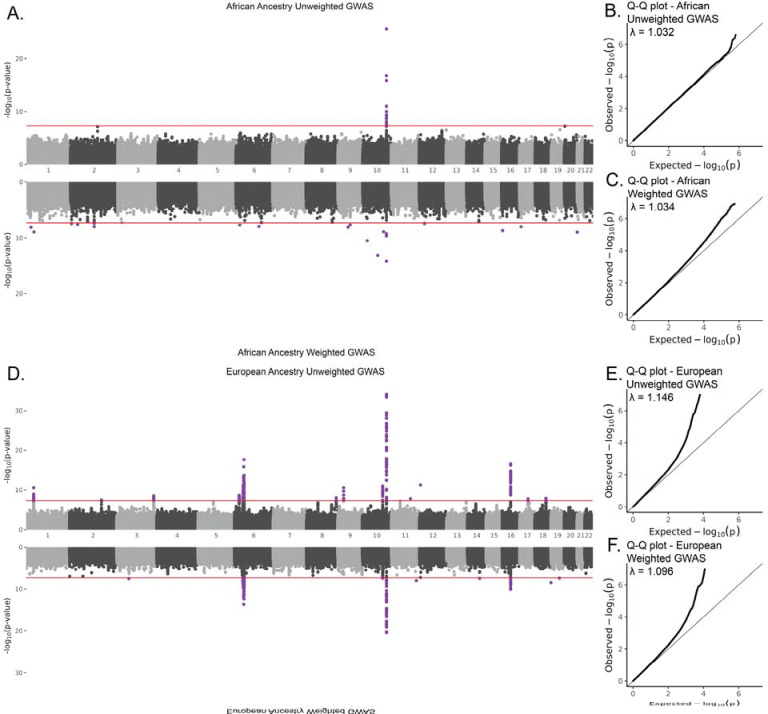
Type 2 diabetes genome-wide association analysis (GWAS). (A) Miami plot comparing the results of a unweighted (top) and weighted (bottom) GWAS for the African ancestry cohort. (B,C) Q-Q plots for African ancestry unweighted and weighted GWAS. (D) Miami plot comparing the results of a unweighted (top) and weighted (bottom) GWAS for the European ancestry cohort. (E,F) Q-Q plots for European ancestry unweighted and weighted GWAS.

We performed GWAS for type 2 diabetes of unweighted and weighted, African and European ancestry cohorts to evaluate the impact of *All of Us* participant IP weighting on genetic associations within and between ancestry groups. The unweighted African ancestry GWAS cohort had 6,481 type 2 diabetes diagnosed cases and 15,138 controls for a total sample size of n=21,619. The effective sample size for the weighted African ancestry GWAS was reduced to n_eff_=9,217. Type 2 diabetes genetic associations for the unweighted and weighted African ancestry cohorts are shown in Figure 3A, with Q-Q plots shown in Figures 3B and 3C. For the unweighted African ancestry GWAS, there is a single peak of 20 genome-wide significant variants (*P*<5×10^−8^) on chromosome 10 in the *TCF7L2* gene, which encodes a transcription factor that regulates genes involved in lipid and glucose metabolism. There are also 20 genome-wide significant variants in the weighted African ancestry GWAS, but they are dispersed across nine different chromosomes. Summary statistics from both the unweighted and weighted GWAS for the African ancestry cohort can be found in Supplementary Table 4. The genomic inflation factors (λ) were close to one for both African ancestry GWAS and changed only slightly between the unweighted and weighted GWAS.

The unweighted European ancestry GWAS cohort had 12,515 type 2 diabetes diagnosed cases and 53,721 controls for a total sample size of n=66,236. The effective sample size for the weighted European ancestry GWAS was n_eff_=35,225. Type 2 diabetes genetic associations for the unweighted and weighted European ancestry cohorts are shown in Figure 3D, with Q-Q plots shown in Figures 3E and 3F. For the unweighted European ancestry GWAS, there are 10 peaks consisting of 1,149 genome-wide significant variants across 10 chromosomes, with two additional significant variants that don’t map to any peak. There are 594 significant variants in the weighted European ancestry GWAS, corresponding to six peaks across five chromosomes, with two additional significant variants that don’t map to any peak. Summary statistics from both the unweighted and weighted GWAS for the European ancestry cohort can be found in Supplementary Table 5. The genomic inflation factors (λ) for both European ancestry GWAS were close to one and slightly reduced in the weighted GWAS.

**Figure 4.**
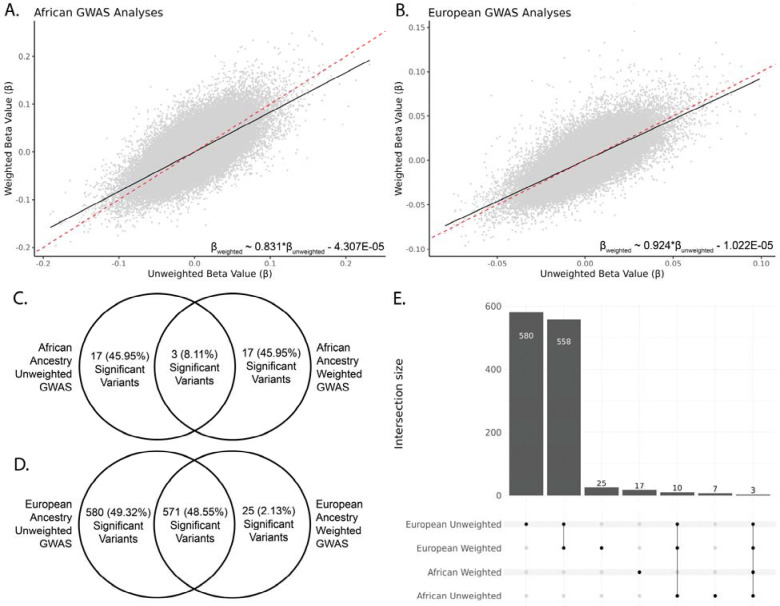
Comparison of African and European ancestry type 2 diabetes genome-wide association analysis (GWAS). (A,B) Show the effect of weighting on variant effect size estimates (Beta values) for both African and European ancestry GWAS. (C,D) Show the number and proportion of variants significant in unweighted and weighted GWAS or both, for African and European ancestry. (E) UpSet plot showing the intersection of genome-wide significant variants between all four GWAS.

We compared how IP weighting changed type 2 diabetes genetic associations for the African and European ancestry *All of Us* participant cohorts. Regression of unweighted and weighted GWAS effect size estimates (β-values), for both African and European ancestry cohorts, shows that they are highly correlated and that IP weighting systematically reduces variant effect sizes (Figure 4A and 4B). Effect sizes are reduced slightly more in the weighted African ancestry cohort (β=0.83) compared to the European ancestry cohort (β=0.94). Similar regressions were performed for variant effect size standard errors and p-values, both of which are highly correlated between unweighted and weighted GWAS (Supplementary Figure 7). For both ancestries, effect size standard errors are higher for the weighted GWAS and −log_10_(*P*-values) are lower for the weighted GWAS, consistent with smaller sample sizes and smaller effect sizes in the weighted GWAS.

For the African ancestry GWAS, there are only 3 out of a total of 37 (8.11%) genome-wide significant variants that were found in both the unweighted and weighted GWAS, and there were 17 unique significant variants in both the unweighted and weighted GWAS (Figure 4C). All three of the significant variants found in both the unweighted and weighted African ancestry GWAS correspond to the same chromosome 10 peak at the *TCF7L2* gene. For the European ancestry GWAS, there are 571 out of a total of 1,176 (48.55%) genome-wide significant variants that were found in both the unweighted and weighted GWAS (Figure 4D). The unweighted European ancestry GWAS has far more unique significant variants (580, 49.32%) compared to the weighted GWAS (25, 2.13%). There are three genome-wide significant variants that are common among all four GWAS, all of which are found in a single peak on chromosome 10. There are 3 variants (rs34872471, rs35198068, rs7903146) that were common among all 4 analyses, all on chromosome 10 (Figure 4E). rs34872471 and rs35198068 have been associated with type 2 diabetes.^12–14^ rs7903146 has been identified as a likely risk allele for type 2 diabetes in ClinVar (RCV002259421.3).^15^

## Discussion

The *All of Us* cohort has become a valuable resource for large-scale genetic epidemiology studies on diverse participant cohorts.^1,3,4^ Nevertheless, the extent to which the *All of Us* participant cohort reflects the demographic, social, lifestyle, and health outcome characteristics of the broader US population has yet to be systematically evaluated. In this study, we showed that the *All of* Us cohort differs substantially from the US population owing to volunteer participant bias. Similar to what has been seen for the UK Biobank, *All of* Us participants tend to be older, more educated, and more female than the US population.^8^ However, the *All of Us* cohort does not have the same healthy volunteer bias that has been observed for other biobank cohorts.^1^ On the contrary, the *All of Us* cohort shows higher prevalence values for a wide variety of disease categories compared to the US population.^1^ Taken together, these findings suggest that the results of disease association studies conducted on the *All of Us* cohort may not be externally valid for the US population.

In light of this problem, we developed a set of inverse probability (IP) weights to increase the population representativeness of the *All of Us* cohort, thereby supporting the external validity of epidemiological studies conducted using the database. Application of these IP weights to the *All of Us* cohort greatly reduced the observed participation bias and brought the demographic, social, and lifestyle characteristics in line with the US population. IP weights also moved the *All of Us* cohort disease prevalence estimates closer to those seen for the US population; although, most prevalence estimates for the weighted *All of Us* cohort remain higher than the US population. Comparison of weighted and unweighted *All of Us* cohorts for type 2 diabetes GWAS, in African and European ancestry cohorts, underscore the extent to which a lack of population representativeness can affect estimated genetic associations with disease.

While the weights we developed are effective in reducing the participation bias in the *All of Us* cohort, there are important limitations to consider. The IP weights we developed were limited to matching based on nine participant characteristics that were common to, and could be harmonized between, the nationally representative NHANES cohort and the *All of Us* cohort. There may other variables that affect participation within the *All of Us* research program and are unaccounted for by our study. In addition, the use of LASSO regression in developing weights limited the size of the cohort that we could use to develop IP weights, since we could only include individuals with complete data. We were able to develop IP weights for 312,210 *All of Us* participants, which corresponds to 76% of the Controlled Tier Dataset version 7. Finally, when considering weight development, there is discussion on whether IP weighting is the best method to use.^16^ Participants with every low or very high probability participation will receive very high and low weights, respectively, which means that their attributes may dominate the weighted estimates. However, our weights tended to match that of the similar study done in the UKBB.^9^

In conclusion, our study reveals the extent of volunteer participant bias in the *All of Us* cohort, while providing one potential solution in the form of IP weights that can be used to mitigate this bias. The IP weights we develop here are made available as a community resource on the *All of Us* Researcher Workbench. Future studies of volunteer participant bias in the *All of Us* cohort could consider other participant characteristics and/or apply different weighting schemes. As the *All of Us* cohort grows over time, and as the research community continues to work on the valuable data therein, it will be important to provide weights of this kind as a way to increase confidence in the external validity of their findings.

## Methods

### Study Cohort Generation

#### All of Us

The *All of Us* Research Program is a large-scale biobank resource collecting demographic, social, and genetic factors for adults within the United States.^1–4^ The *All of Us* Research Program is comprised of volunteers from diverse backgrounds. Currently, the program stands at over 815,000 participants, with a goal of recruiting over 1 million participants. We initially included all participants with demographic and survey data in the initial cohort (n=379,454). This cohort was built using version 7 of the *All of Us* Controlled Tier Dataset.

#### National Health and Nutrition Examination Survey (NHANES)

The National Health and Nutrition Examination Survey (NHANES) is a program run by the National Center for Health Statistics (NCHS), a part of the Centers for Disease Control and Prevention (CDC),^10^ to assess the health and nutritional status of adults and children within the United States. The survey examines a nationally representative sample of about 5,000 participants each year. NHANES collects demographic, socioeconomic, dietary, and health-related questions. We used the 2017 – March 2020 NHANES sample, and initially included all participants within the NHANES cohort (n=15,560).

#### Variable Harmonization

To match variables between the *All of Us* and NHANES participant datasets, we harmonized common variables such that the questions and the responses used by the two databases showed a one-to-one correspondence. We were able to successfully harmonize nine variables common to the *All of Us* and NHANES datasets. The original and harmonized questions and answers can be seen in Supplementary Table 1.

### Inverse Probability (IP) Weight Generation

#### Participant Variables

To develop weights, we initially chose eleven variables for participant characteristics that were common to the NHANES and *All of Us* cohorts. We removed height and weight measurements due to missingness greater than 15% within the *All of Us* cohort, leaving nine common variables. From there, we subsetted each of the cohorts such that the age ranged from 18 to 79 and removed participants with missing race. The final variables included in the model can be viewed in Supplementary Table 1. Finally, LASSO regression requires participants to have no missing data, yielding 7,430 complete cases within the NHANES cohort, and 312,210 complete cases within the *All of Us* cohort. The full cohort creation for *All of* Us and NHANES can be viewed in Supplementary Figures 1 and 2. The final nine participant variables included in the model were: age, self-identified race and ethnicity (SIRE), gender, birthplace, highest grade, marital status, health insurance, smoking habits, and drinking habits. Age was coded numerically, while all other variables were coded categorically.

#### IP Weight Construction

We combined the harmonized NHANES and *All of Us* datasets described above to construct IP weights for the *All of Us* cohort. We used a logistic LASSO regression in glmnet^17^ to predict the probability of *All of Us* participation for each participant based on the nine variables included in the model. We coded *All of Us* participants with 1, and NHANES participants with a 0. We included weights from the NHANES study. Age was included as a numeric variable, and all other variables were coded categorically and converted to dummy variables using fastDummies^18^. To predict weights, we included each possible two-way interaction terms among the dummy and continuous variables. LASSO performs variable selection to include the predictors that contribute the most to participation.

Using the LASSO model described above, we predicted participation probability $\left(P_{i}\right)$ for each individual participant within the *All of Us* cohort. Using this probability, we calculated the raw IP weight for each individual participant $\left(w_{i}\right)$ using the following formula: $w_{i}=\frac{1-P_{i}}{P_{i}}$, followed by mean normalization of the raw IP weights.

### IP Weight Validation

#### Population Mean and Proportion Recovery

We initially validated the performance of the IP weights by measuring the recovery of the nationally representative NHANES population means and proportions for the nine variables, with the weights applied to the *All of Us* cohort. Using the following formula, $\frac{\sum_{i=1}^{n}\;w_{i}X_{i}}{\sum_{i=1}^{n}\;w_{i}}$, we predicted weighted means and proportions for the *All of Us* cohort to compare against that of NHANES.

To further evaluate performance, we measured if the weights developed reduced disease prevalence. Denny et. al. demonstrated that the *All of Us* cohort, unlike other prominent biobanks, has an overrepresentation of disease diagnosed individuals.^1^ Using data from the 2019 Global Burden of Disease (2019 GBD) study,^11^ we found that our cohort had an overrepresentation of diseased individuals relative to the US population. We then found weighted prevalences for the same disease categories and compared them to that of the 2019 GBD.

#### Correlation Coefficients

To measure the reduction in participation bias, we measured the differences in correlation coefficients between all the nine variables in the NHANES cohort, *All of Us* cohort (both unweighted and weighted). We quantified the reduction in participation bias as the degree to which the *All of Us* weighted correlation coefficient moved closer to the NHANES correlation coefficient. We measured the correlation between numeric and categorical variables using point biserial correlation, and the correlation between categorical variables using Cramer’s V.

### Weighted GWAS

To further quantify the effects of participation bias on genetic studies within the *All of Us* cohort, we conducted GWAS studies on both African and European ancestry cohorts for type 2 diabetes. We used the LDAK (version 5.2)^19^ package to conduct both unweighted and weighted GWAS studies for both ancestry cohorts. These models included the covariates (PC1-PC5, sex, and age). We obtained unweighted and weighted variant effect size estimates, standard errors, and p-values for all GWAS.

To address the loss of precision when using weighting in genomic analyses, we calculated effective samples sizes for the European and African ancestry cohorts using the following formula: $n_{eff}=\frac{{\left(\sum w_{i}\right)}^{2}}{\sum w_{i}^{2}}$

## Supplementary Material

Supplement 1

Supplement 2

## Figures and Tables

**Table 1.** *All of Us* and NHANES participant comparison

Characteristic		All of Us	SD/n	NHANES	SD/n	Delta

n		312,210		7,430		
Age		53.91	15.55	46.99	16.31	6.92
Race	White	56.22%	175518	62.90%	2451	−6.68%
	Black	18.52%	57827	11.35%	2041	7.17%
	Hispanic/Latino	19.15%	59774	16.13%	1698	3.02%
	Asian	3.65%	11397	5.57%	875	−1.92%
	Other	2.46%	7694	4.06%	365	−1.60%
Gender	Male	36.75%	114749	48.86%	3634	−12.11%
	Female	63.25%	197461	51.14%	3796	12.11%
Birthplace	Born in US	84.26%	263075	81.11%	5278	3.15%
	Other	15.74%	49135	18.89%	2152	−3.15%
Highest Grade	Less than 9th Grade	3.16%	9854	3.25%	520	−0.09%
	9–11th Grade	5.71%	17831	6.82%	778	−1.11%
	High school graduate/GED or equivalent	18.53%	57841	26.93%	1802	−8.40%
	Some college or AA degree	26.22%	81874	30.80%	2478	−4.58%
	College graduate or above	46.38%	144810	32.22%	1852	14.16%
Marital Status	Married/Living with Partner	52.09%	162640	62.93%	4355	−10.84%
	Widowed/Divorced/Separated	21.48%	67073	17.04%	1536	4.44%
	Never Married	26.42%	82497	20.03%	1539	6.39%
Covered by Health Insurance	No	6.72%	20979	13.72%	1255	−7.00%
	Yes	93.28%	291231	86.28%	6175	7.00%
Smoking Habits	Never smoked	61.81%	192968	57.17%	4321	4.64%
	Former smoker	21.88%	68325	25.31%	1682	−3.43%
	Sometimes smokes	5.19%	16193	4.01%	321	1.18%
	Daily smoker	11.12%	34724	13.51%	1106	−2.39%
Alcohol Habits	Never drank	10.04%	31345	6.47%	666	3.57%
	Less than yearly	15.66%	48879	15.16%	1393	0.50%
	Monthly or less	30.86%	96356	31.74%	2388	−0.88%
	Two to four times a month	19.66%	61376	22.54%	1477	−2.88%
	Twice or more a week	23.78%	74254	24.09%	1506	−0.31%

## Data Availability

The nationally representative database used to develop weights was the 2017 – March 2020 National Health and Nutrition Examination Survey (NHANES). This data is free to access and publicly available at: https://wwwn.cdc.gov/nchs/nhanes/continuousnhanes/default.aspx?Cycle=2017-2020 We used version 7 of the *All of Us* Controlled Tier Dataset, which can be accessed and analyzed from the Researcher Workbench by registered users: https://www.researchallofus.org/data-tools/workbench/
