# Supplementary material for: Increasing Representativeness in the All of Us Cohort Using Inverse Probability Weighting
Source: medRxiv. 2024 Oct 2:2024.10.02.24314774. Preprint. [Version 1] doi: 10.1101/2024.10.02.24314774 (PMC11722450; doi:10.1101/2024.10.02.24314774)
Supplement: Supplementary file 1 [file media-1.docx]

**Increasing Representativeness in the** ***All of Us*** **Cohort Using Inverse Probability Weighting**

Manoj S. Kambara^1^, Shivam Sharma^2,3^, John L. Spouge^4^, I. King Jordan^2,3^, Leonardo Mariño-Ramírez^1,*^

^1^National Institute on Minority Health and Health Disparities, National Institutes of Health, Bethesda, Maryland, USA

^2^IHRC-Georgia Tech Applied Bioinformatics Laboratory, Atlanta, Georgia, USA

^3^School of Biological Sciences, Georgia Institute of Technology, Atlanta, Georgia, USA

^4^National Center for Biotechnology Information, National Library of Medicine, National Institutes of Health, Bethesda, Maryland, USA

**Contents**

**Supplementary Methods**	2

**1.1**	**Cohort creation and genomic data analysis**	2

**Supplementary Figures**	3

**Supplementary Figure 1. NHANES participant cohort creation.**	3

**Supplementary Figure 2. All of Us participant cohort creation.**	4

**Supplementary Figure 3. Graphs for LASSO regression model used for IP weight development.**	5

**Supplementary Figure 4. Mean and proportion recovery for remaining auxiliary variables.**	6

**Supplementary Figure 5. Disease prevalence estimates for males in both the US and unweighted and weighted** ***All of Us*** **cohort.**	7

**Supplementary Figure 6. Disease prevalence estimates for females in both the US and unweighted and weighted** ***All of Us*** **cohort.**	8

**Supplementary Figure 7. Standard errors and p-values for standard and weighted European and African ancestry GWAS.**	9

# **Supplementary Methods**

## 1.1 **Cohort creation and genomic data analysis** 

The study cohort was built from the *All of Us* Controlled Tier dataset v7 (curated version C2022Q4R9), which includes participants enrolled from 2018-2022, with a data cutoff date of 7/1/2022.  Participants who self-identified as American Indian or Alaska Native were not included in the analysis. 

Genetic ancestry for the *All of Us* participants were inferred using supervised clustering, as implemented in Rye[^1], on the genotyping array data. We created two cohorts with majority African or European ancestries using the genetic ancestry estimates and predicted categorical ancestry labels provided by the *All of Us* Research Program. Participants with >50% African ancestry and predicted categorical ancestry label of AFR (African/African American) were included in the African ancestry cohort. Participants with >50% European ancestry and predicted categorical ancestry label of EUR (European) were included in the European ancestry cohort.

Genome-wide association analyses were run on a smaller call set extracted from short read Whole Genome Sequencing data (srWGS). This call set, which is based on Allele Count/Allele Frequency (ACAF) thresholds (AF > 1%; AC > 100), is provided in the *All of Us* CDR directory (v7.1).  Cohort specific QC was subsequently performed using Plink v2[^2] and variants with >10% missingness, AF < 1%, and AC < 50 were removed. A Hardy-Weinberg equilibrium (HWE) filter with threshold set to 10^-6^ was further applied to remove variants with HWE exact test p-values below the threshold.

# **Supplementary Figures**

**Supplementary Figure 1. NHANES participant cohort creation.** 

Inclusion criteria and the number of participants retained at each step. 

**Supplementary Figure 2. All of Us participant cohort creation.** 

Inclusion criteria and the number of participants retained at each step. 

## **Supplementary Figure 3. Graphs for LASSO regression model used for IP weight development.** 

These graphs show the process of variable selection by the LASSO regression algorithm.

## **Supplementary** **Figure 4. Mean and proportion recovery for remaining auxiliary variables.** 

For the variables, birthplace, health insurance, marital status, alcohol habits, and smoking habits, the IP weights we developed are still effective in recovering population proportions.

## **Supplementary** **Figure 5. Disease prevalence estimates for males in both the US and unweighted and weighted** ***All of Us*** **cohort.** 

Disease prevalence estimates for 14 broad disease categories are shown for the US population, estimated from GBD2019 data (green stars), the unweighted *All of Us* cohort (blue circles), and the weighted *All of Us* cohort (orange circles). For 10 disease categories, we see that the IP weights are effective in bringing disease prevalence in *All of Us* closer to the national prevalence.

## **Supplementary** **Figure 6. Disease prevalence estimates for females in both the US and unweighted and weighted** ***All of Us*** **cohort.**

Disease prevalence estimates for 15 broad disease categories are shown for the US population, estimated from GBD2019 data (green stars), the unweighted *All of Us* cohort (blue circles), and the weighted *All of Us* cohort (orange circles). For 9 disease categories, we see that the IP weights are effective in bringing disease prevalence in *All of Us* closer to the national prevalence.

## **Supplementary** **Figure 7. Standard errors and p-values for standard and weighted European and African ancestry GWAS.**

(A) Shows how there is a systematic increase in standard error when applying IP weights to European ancestry GWAS. (B) Shows a systematic decrease in the -log_10_(p-value) when applying IP weights to European ancestry GWAS. (C) Shows how there is a systematic increase in standard error when applying IP weights to African ancestry GWAS. (D) Shows a systematic decrease in the -log_10_(p-value) when applying IP weights to African ancestry GWAS.

[^1]: Conley, A.B. et al. Rye: genetic ancestry inference at biobank scale. Nucleic Acids Res. 51, e44 (2023)
[^2]: Chang, C.C. et al. Second-generation PLINK: rising to the challenge of larger and richer datasets. Gigascience 4, 7 (2015)
